# Covid-19 and impulsivity: an evaluation of self-harm admissions in emergency care

**DOI:** 10.1192/j.eurpsy.2022.335

**Published:** 2022-09-01

**Authors:** A. Mariano, R. Santini, T. Jannini, F. Di Michele, F. Bianchi, A. Siracusano, C. Niolu

**Affiliations:** University of Rome Tor Vergata, Department Of System Medicine, Rome, Italy

**Keywords:** emergency care, self-harm, Impulsivity, Covid-19

## Abstract

**Introduction:**

Several studies highlighted how COVID-19-related isolation and quarantine deeply weighed on the mental health of both the general and psychiatric population. There has been limited investigation about self-harm and impulsivity during the COVID-19 pandemic.

**Objectives:**

The aim of this study is to evaluate how COVID-19-related lockdown affected self-harm rates in an Italian hospital.

**Methods:**

Data on 59 patients were retrospectively collected from the Emercency deparment of the Policlinico Tor Vergata, Rome, from March 11 to May 4, 2020 (Italian mass quarantine) and the same periods of 2019 and 2021. Demographics, psychiatric history, substance use/abuse, types of self-harm and admission in psychiatric acute unit (PAU) rates were recorded.

**Results:**

No statistical difference was reported in self-harm rates [9.8%(26/266) in 2019 vs 13.2%(10/76) in 2020 vs 10.7%(23/215) in 2021;p>0.05]. In 2020 subjects were younger (31.9±12.1 vs 39.2±14.4,p=0.22;vs 38.1±14.4;p=0.15) and had higher incidence of psychiatric history [90%(9/10) vs 73.1%(19/26), p=0.42;vs 65.2% (15/23),p=0.29],than 2019 and 2021 respectively. Substance use/abuse rates were significantly lower in 2020 compared to 2019 and 2021 [10%(1/10) vs 53.8%(14/26),p=0.04;vs 60.9% (14/23), p=0.02]. In 2020, subjects committing self-harms were more frequently admitted to PAU compared to 2019 and 2021 [60%(6/10)vs19.2%(5/26),p=0.04; vs 17.4% (4/23), p=0.04).

**Conclusions:**

Consistent with the literature, lockdown-related measures negatively impacted on younger people, with higher rates of self-harm between March and May 2020. This, together with a higher rate of admissions to PAU, should warn the mental health system to target with specific programs to support adolescents and youngers.

**Disclosure:**

No significant relationships.

